# Complete mitogenomes of the marine picoplanktonic green algae *Prasinoderma* sp. MBIC 10622 and *Prasinococcus capsulatus* CCMP 1194 (Palmophyllophyceae)

**DOI:** 10.1080/23802359.2019.1698370

**Published:** 2019-12-11

**Authors:** Monique Turmel, Christian Otis, Claude Lemieux

**Affiliations:** Département de biochimie, de microbiologie et de bio-informatique, Institut de Biologie Intégrative et des Systèmes, Université Laval, Québec, Canada

**Keywords:** Chlorophyta, mitogenome evolution, phylogenomics, Prasinococcales, *trans*-spliced intron

## Abstract

Marine picoalgae from the Prasinococcales order occupy the deepest branch of the Chlorophyta (Palmophyllophyceae). Here, we describe the mitogenomes of *Prasinoderma* sp. MBIC 10622 and *Prasinococcus capsulatus* CCMP 1194. At 37,590 and 41,006 bp, respectively, they are smaller than their *Prasinoderma coloniale* homolog and unlike the latter, lack an inverted repeat. The intronless *Prasinoderma* sp. mitogenome possesses the largest gene repertoire (68) among all chlorophytes examined to date. At the gene order level, it displays more ancestral traits than its prasinococcalean homologs, closely resembling the mitogenomes of Mamiellophyceae. Remarkably, the *P. capsulatus* mitogenome features a *trans*-spliced group II intron.

The recently erected class Palmophyllophyceae, which comprises the prasinophyte orders Palmophyllales (three genera) and Prasinococcales (two genera), represents the deepest-branching lineage of the Chlorophyta (Leliaert et al. 2016). Given this phylogenetic position, analyses of organelle genomes from the Palmophyllophyceae may offer valuable insights into the genome architecture of the first chlorophytes. Although the complete plastomes of four prasinophytes belonging to the aforementioned orders are publicly available (Lemieux et al. 2014; Leliaert et al. 2016), a single mitogenome (that of *Prasinoderma coloniale*), is currently available for the Palmophyllophyceae (Pombert et al. 2013). Here, we describe the mitogenomes of two additional representatives of the Prasinococcales, *Prasinoderma* sp. and *Prasinococcus capsulatus.*

The strain of *Prasinoderma* sp. (NBRC 102842, formally MBIC 10622) was obtained from the Biological Resource Center of the National Institute of Technology and Evaluation (Chiba, Japan), whereas the *P. capsulatus* strain (CCMP 1194) was obtained from the Bigelow National Center for Marine Algae and Microbiota (Maine, USA). For each strain, an A + T-rich organellar DNA fraction was subjected to 454 GS-FLX DNA Titanium pyrosequencing, the resulting reads were used to assemble the plastome and mitogenome, and organelle genes were annotated as described (Lemieux et al. 2014).

The *Prasinoderma* sp. and *P. capsulatus* mitogenomes were assembled as circular molecules of 37,590 bp (GenBank MN662311) and 41,006 bp (GenBank MN662312), respectively. They are smaller than their *P. coloniale* homolog (54,546 bp) and unlike the latter, they exhibit no inverted repeat, a feature thought to be ancestral (Pombert et al. 2013; Turmel et al. 2019). Despite its smaller size, the *Prasinoderma* sp. mitogenome encodes 7 and 13 extra genes compared to the *P. capsulatus* and *P. coloniale* mitogenomes. Its repertoire of 68 conserved genes is the largest among all chlorophytes examined thus far and includes the previously unidentified *rps1* and *sdh4* genes. At the gene order level, it shows more similarity with the mitogenomes of Mamiellophyceae (Mamiellales) than those of Prasinococcales. For example, the 17.9-KB segment extending from *nad7* to *rnl* (38 genes) is colinear with the *Micromonas commoda* and *Ostreococcus tauri* mitogenomes (Robbens et al. 2007; Worden et al. 2009).

No mitochondrial introns are present in *Prasinoderma* sp., whereas two are found in *P. capsulatus*: a *cis*-spliced group I intron encoding a LAGLIDADG homing endonuclease within *rnl* and a *trans*-spliced group II intron encoding a reverse transcriptase/intron maturase within *cox1*. The breakpoint of the latter intron maps to domain IV of the intron secondary structure. *Trans*-spliced introns occur rarely in chlorophyte mitochondria but seem to be more prevalent in the Palmophyllophyceae, as two *trans*-spliced group I introns were uncovered in the *P. coloniale rnl* gene (Pombert et al. 2013).

A maximum-likelihood phylogeny was inferred from 32 concatenated mitogenome-encoded proteins of 22 chlorophytes and nine streptophyte green algae using RAxML v.8.2.3 (Stamatakis 2014). The best-scoring tree shows that the prasinococcalean taxa cluster together, with *Prasinoderma* sp. forming a short branch and the two others much longer branches (Figure 1). The *Prasinoderma* species were not recovered as a monophyletic group possibly due to long-branch attraction.

**Figure 1.**
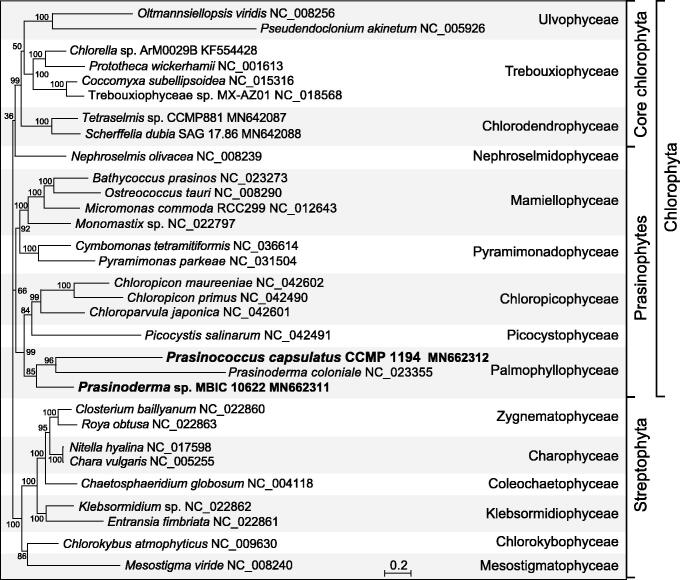
RAxML analysis of 32 concatenated mitogenome-encoded proteins from 22 chlorophytes and nine streptophyte green algae. The figure shows the best-scoring tree, with the bootstrap support values (100 replicates) reported on the nodes. GenBank accession numbers are provided for the mitogenomes of all taxa. The scale bar denotes the estimated number of amino acid substitutions per site. The data set was generated using the predicted protein sequences derived from the following genes: *atp1*, *4*, *6*, *8*, *9*, *cob*, *cox1*, *2*, *3*, *mttB*, *nad1*, *2*, *3*, *4*, *4L*, *5*, *6*, *7*, *9*, *rpl5*, *6*, *16*, *rps2*, *3*, *4*, *7*, *10*, *11*, *12*, *13*, *14*, and *19*. Following alignment of the sequences of individual proteins with Muscle v3.7 (Edgar 2004), ambiguously aligned regions were removed using TrimAL v1.4 (Capella-Gutierrez et al. 2009) with the options block = 6, gt = 0.7, st = 0.005, and sw = 3, and the protein alignments were concatenated using Phyutility v2.2.6 (Smith and Dunn 2008). For the phylogenetic analysis, the data set was partitioned by protein and the GTR + Γ4 model was applied to each of the 32 partitions.
